# From Rest to Growth: Life Collisions of *Gordonia polyisoprenivorans* 135

**DOI:** 10.3390/microorganisms10020465

**Published:** 2022-02-18

**Authors:** Nataliya E. Suzina, Vladimir V. Sorokin, Valentina N. Polivtseva, Violetta V. Klyueva, Elena V. Emelyanova, Inna P. Solyanikova

**Affiliations:** 1 Federal Research Center “Pushchino Scientific Center for Biological Research of the Russian Academy of Sciences”, Institute of Biochemistry and Physiology of Microorganisms, 142290 Pushchino, Russia; suzina_nataliya@rambler.ru (N.E.S.); kaistia@gmail.com (V.N.P.); elenvem@ibpm.pushchino.ru (E.V.E.); 2 Federal Research Center of Biotechnology of the Russian Academy of Sciences, Winogradsky Institute of Microbiology, 117312 Moscow, Russia; vlvlsorokin@gmail.com; 3 Institute of Pharmacy, Chemistry and Biology, Regional Microbiological Center, Department of Biotechnology and Microbiology, Belgorod National Research University, 308015 Belgorod, Russia; klyueva@bsu.edu.ru

**Keywords:** *Gordonia polyisoprenivorans* 135, dormancy, germination, ultrathin organization, acidocalcisomas, degradation activity

## Abstract

In the process of evolution, living organisms develop mechanisms for population preservation to survive in unfavorable conditions. Spores and cysts are the most obvious examples of dormant forms in microorganisms. Non-spore-forming bacteria are also capable of surviving in unfavorable conditions, but the patterns of their behavior and adaptive reactions have been studied in less detail compared to spore-forming organisms. The purpose of this work was to study the features of transition from dormancy to active vegetative growth in one of the non-spore-forming bacteria, *Gordonia polisoprenivorans* 135, which is known as a destructor of such aromatic compounds as benzoate, 3-chlorobenzoate, and phenol. It was shown that *G. polyisoprenivorans* 135 under unfavorable conditions forms cyst-like cells with increased thermal resistance. Storage for two years does not lead to complete cell death. When the cells were transferred to fresh nutrient medium, visible growth was observed after 3 h. Immobilized cells stored at 4 °C for at least 10 months regenerated their metabolic activity after only 30 min of aeration. A study of the ultrathin organization of resting cells by transmission electron microscopy combined with X-ray microanalysis revealed intracytoplasmic electron-dense spherical membrane ultrastructures with significant similarity to previously described acidocalcisomas. The ability of some resting *G. polyisoprenivorans* 135 cells in the population to secrete acidocalcisome-like ultrastructures into the extracellular space was also detected. These structures contain predominantly calcium (Ca) and, to a lesser extent, phosphorus (P), and are likely to serve as depots of vital macronutrients to maintain cell viability during resting and provide a quick transition to a metabolically active state under favorable conditions. The study revealed the features of transitions from active growth to dormant state and vice versa of non-spore-forming bacteria *G. polyisoprenivorans* 135 and the possibility to use them as the basis of biopreparations with a long shelf life.

## 1. Introduction

Members of the genus *Gordonia* (phylum Actinobacteria, order Actinomycetales, suborder Corynebacterineae) are aerobic, catalase-positive, Gram-positive to Gram-variable, slightly acid-fast, nonmotile, nocardioform actinomycetes [1]. They are aerobic heterotrophs, containing mycolic acid-rich cell walls, and have been isolated from terrestrial and aquatic sources, including hydrocarbon-contaminated industrial sites. Despite some of them having been implicated in opportunistic infections in immunocompromised individuals and foaming in wastewater treatment plants [1,2], representatives of the genus *Gordonia* are undoubtedly important in the natural environment and, due to their ability to degrade substituted and unsubstituted hydrocarbons, they are powerful candidates for bioremediation processes. *Gordonia* sp. strain QH-12 isolated from activated sludge was shown “to be capable of utilizing dibutyl phthalate and other common phthalate esters” [3]. *Gordonia rubripertincta* CWB2 was shown to degrade styrene [4], while *Gordonia alkanivorans* DSM 44369^T^ degraded alkanes [5], *G. amicalis* DSM 44461^T^ was active against dibenzothiophene [6,7], and *Gordonia* sp. strain TY-5 utilized propane and acetone. *Gordonia* sp. i37 was among the isolated bacteria with the ability to degrade isoprene [8]. Thus, the ability to oxidize a number of persistent compounds makes the members of the genus *Gordonia* promising and preferential candidates in biopreparations.

As well as biodegradative activity, *Gordonia* spp. cells can synthesize a number of technologically significant metabolites and enzymes. *Gordonia* can also be considered as producers of valuable compounds, among them gordonan, synthesized only by these bacteria [1], and some biosurfactants [9].

Sowani et al. presented several papers devoted to the individual properties of *Gordonia* cells and an extensive review on the decomposition of various pollutants by *Gordonia*, including hydrocarbons, phthalates, rubber, nitriles, explosives, and the desulfurization of sulfur compounds, highlighting the interaction of cells with metals and their role in wastewater treatment processes [10,11,12]. Based on the available data, it can be unambiguously concluded that *Gordonia* are promising candidates for the creation of effective biological products on their basis.

In a review devoted to the characterization of the *Gordonia*, Drzyzga [13] summarized the potential of representatives of this genus for ecology and biotechnological industry, noting it as a “strength” of the genus. Additionally, as a “weakness”, he defined the possible pathogenic effect on humans [13,14]. Regardless of whether *Gordonia* act as pathogens or as biotechnologically significant agents, the importance of microorganisms belonging to this group is very great. However, there is practically no discussion about how *Gordonia* survive adverse conditions, including cell behavior during storage, the possibility and rate of return to a metabolically active state, as well as several related issues that are important for the development and successful use of biological products based on these cells. Among these questions, the peculiarity of cells’ transition between the physiologically active state and the state of rest remains poorly studied. The ability to survive unfavorable factors has been studied in detail in microorganisms that form special surviving forms, such as cysts and spores [15,16,17,18,19,20,21,22]. However, for non-spore-forming microorganisms, it has been shown that, when nutrients are depleted, with a nitrogen limit, under the action of autoregulators, they are able to form specific surviving forms, named cyst-like resting cells (CLC).

Cyst-like cells of non-spore-forming microorganisms can be considered as a form of survival under unfavorable conditions. It is known that, along with true spores of spore-forming bacteria, CLCs are characterized by increased resistance to damaging influences, can maintain the ability to resume growth for a long time, and the germination of CLCs leads to the appearance of morpho-physiological differences between the colonies being formed. These differences are manifested in the rate of cell growth and their metabolic activity. The ability to form CLC has been shown for a few bacterial genera [23,24,25,26].

The *G. polyisoprenivorans* strain 135 was shown to degrade several resistant pollutants, such as 3-chlorobenzoic acid and chlorophenols [27]. Due to the rather narrow substrate specificity of both benzoate 1,2-dioxygenase and catechol 1,2-dioxygenase, cells of the 135 strain have been proposed for use as a biosensor for benzoate detection [28]. The successful use of cells, both immobilized and stored in suspension, requires an understanding of the processes that occur in cells during long-term storage. The aim of this work was to study the ability of *G. polyisoprenivorans* strain 135 to survive under unfavorable cultivation conditions, such as the depletion of nitrogen and carbon, and to renew the growth under optimal ones.

## 2. Material and Methods

### 2.1. Bacterial Culture and Cultivation Conditions

The bacterial strain *Gordonia polyisoprenivorans* 135 [27] was used in this work. Cells were grown in mineral CP1 medium with the following composition (g/L): Na_2_HPO_4_, 0.7; KH_2_PO_4_, 0.5; NH_4_NO_3_, 0.75; MgSO_4_ × 7H_2_O, 0.2; MnSO_4_, 0.001; FeSO_4_, 0.02 [29], with sodium benzoate as the sole source of carbon and energy, or in lysogenic broth (LB) containing (g/L): tripton 10; sodium chloride 10; yeast extract 5. To obtain LB agar medium, the broth was supplemented with agar-agar 20 g/L.

### 2.2. Obtaining the Resting Forms

**Figure 1:**
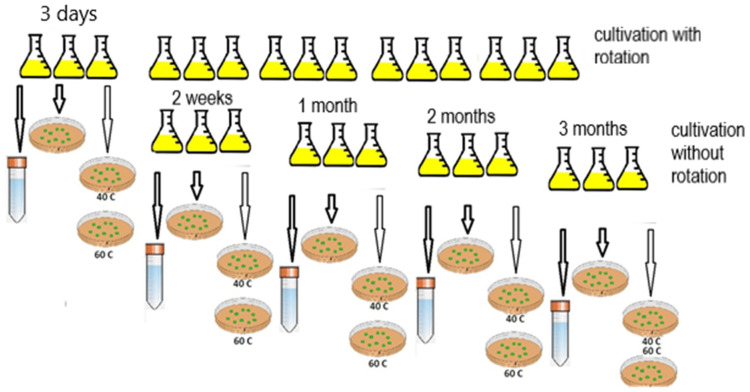
Scheme of obtaining the resting forms of *G. polyisoprenivorans* 135.

The cultivation was carried out at 28 °C on a rotary shaker at 180 rpm for three days, then the cultivation was continued at rest in a thermostat at 23 °C. In parallel, cultivation was performed without the use of a shaker; 15 flasks with LB medium inoculated with the culture were kept at rest in a thermostat at 23 °C. At different time intervals—3 days, 2 weeks, 1 month, 2 months, 3 months, and 6 months—cells were sequentially sampled from the flasks to measure culture growth parameters, as well as to analyze the enzymatic activity (Figure 1). Sampling from the flasks and all measurements were performed in three replicates.

### 2.3. Viability of the Vegetative Cells and CLC

Cell viability was determined by direct colony count (CFU/mL) after the inoculation of dilutions aliquots (10^n^) on LB agar plates and cultivation at 28 °C for 3 days. 

To test the thermal stability of *G*. *polyisoprenivorans* 135 cells, the bacterial suspension in two dilutions, 10^−7^ and 10^−8^, of the original was incubated for 10 min at 40 and 60 °C. Then, 100 μL of the suspension was inoculated onto Petri dishes with LB agar medium.

### 2.4. Microscopically Techniques

#### 2.4.1. Phase Contrast Microscopy

Microscopic observations were carried out using a Nikon Eclipse Ci microscope (Nikon, Japan) equipped with a ProgResSpeedXTcore5 camera (Jenoptik, Germany) and Axioplan (Carl Zeiss, Germany). 

#### 2.4.2. Fluorescence Microscopy 

Bacterial cells were stained with the Live/Dead BacLight Bacterial Viability Kit^®^ L-13152 (Molecular Probes, Eugene, OR, USA) according to the manufacturer’s recommendations. At least 1000 cells were counted in 20–25 fields.

#### 2.4.3. Electron Microscopy 

Cell pellets were concentrated by centrifugation (10,000× *g*, 15 min), fixed with 2% glutaraldehyde in 0.05 M cacodylate buffer (pH 7.2) for 1 h at 4 °C and further treated as described earlier [30]. Ultrathin sections were analyzed under a JEM-1400 transmission electron microscope (JEOL, Musashino, Japan) at 80 kV.

### 2.5. X-ray Microanalysis

X-ray microanalysis of the elemental composition of acidocalcisome-like ultrastructures on thin sections was carried out without additional staining using a JEM-1400 microscope (JEOL, Japan) equipped with an X-ray microanalyzer (Oxford Instruments, United Kingdom) at an accelerating voltage of 80 keV.

### 2.6. Assessment of Cells Metabolic Activity

The ability to maintain metabolic activity under starvation and desiccation stress conditions was assessed by cell survival parameters and their ability to perform benzoate degradation. The biodegradative activity of cells was assessed according to changes in the activity of benzoate 1,2-dioxygenase (BDO) (EC 1.14.12.10). This enzyme is known to initiate the decomposition of benzoate [28]. BDO activity was estimated by the reaction rate with sodium benzoate. The respiratory activity of cells was determined by measuring their O_2_ consumption after resuspension in 50 mM Tris-HCl buffer, pH 7.6. The metabolic activity was indicated by the presence of BDO activity, benzoate degradation enzymes, oxidative stress defense enzymes, and respiratory activity in the cells.

### 2.7. G. polyisoprenivorans 135 Cells Immobilization and Bioreceptor Development

*G. polyisoprenivorans* cells were cultivated on LB agar medium at 28 °C for 18 h. The biomass was washed off the surface of the agar with 50 mM potassium-sodium phosphate buffer, pH 7.4. In the resulting suspension, the concentration of wet cells was 100 mg/mL. Before bioreceptor preparation, cells were incubated for 24 h at 4 °C. The formation of the bioreceptor was carried out according to the previously described method [28].

### 2.8. Polarographic Measurement of Cell Respiration and BDO Activity in Nonimmobilized G. polyisoprenivorans Cells

To determine the *G. polyisoprenivorans* 135 cells respiratory activity and BDO enzymatic activity, biomass grown in liquid mineral medium containing 0.2–0.25 g/L sodium benzoate was pelleted by centrifugation (16,000× *g*, 15 min, 4 °C), suspended in the same buffer, and immediately analyzed. Measurements were carried out according to the previously described method [28]. In this case, the signal reflected the rate of the respiration, expressed as μg O_2_/(L s).

BDO activity in non-immobilized cells was measured by adding benzoate or substituted benzoates to the cell suspension. The measurements were carried out according to the previously described procedure [28]. The reaction rate was expressed as pA/s (1 pA/s ≈ 0.153 μg O_2_/(L s)).

The Clark electrode was used as a part of an amplification system (Ingold 5313/10 O_2_ amplifier; Ingold, Switzerland–United States). The signal registration was performed with a 2-coordinate recorder (XY Recorder-4103; Recorder, Czech Republic).

### 2.9. Measurement of BDO Activity in Immobilized G. polyisoprenivorans Cells

In the case of using cells immobilized on a membrane, the standard procedure for measuring BDO activity did not differ from that described earlier [28].

At the first stage, the basic respiration of immobilized cells was registered. At the second stage, a solution of sodium benzoate or its analogue was introduced into the cuvette. Cell oxygen consumption was measured, which was proportional to BDO activity, since oxygen is the second substrate involved in the benzoate oxidation reaction. The reaction rate of BDO-substrate was expressed in pA/s. 

### 2.10. Obtaining a Cell-Free Extract

Cells grown in liquid LB medium with and without agitation were collected at 3 days, 7 days, 14 days, 1 month, 2 months, 3 months, and 6 months by means of centrifugation at 5000× *g* for 5 min at 4 °C (5804R Eppendorf AG, Hamburg, Germany). Cells were washed with 50 mM Tris/HCl-buffer, pH 7.6 and spun off under the same conditions. The washed cells were destroyed using an automatic mill (PULVERISETTE 23, FRITSCH, Idar-Oberstein, Germany) at 50 power for two minutes. Undiluted cells were removed by centrifugation, and the supernatant was used to determine protein concentration and enzyme activity.

### 2.11. Determination of Enzyme Activity in Cell-Free Extract

Enzyme activities were determined spectrophotometrically on a Shimadzu UV-1900i spectrophotometer (Japan) in quartz cuvettes with an optical path length of 1 cm at 25 °C.

The activity of catechol 1,2-dioxygenase (Cat 1,2-DO) (EC 1.13.11.1) was determined according to Hayaishi [31] with some modifications. 

The activity of catechol 2,3-dioxygenase (Cat 2,3-DO) (EC 1.13.11.2) was determined as described earlier [32].

The activity of catalase (EC 1.11.1.6) in the supernatants was determined as hydrogen peroxide decomposition [33]. Catalase activity was calculated according to H_2_O_2_ molar extinction coefficient ε = 43,600 M^−1^ cm^−1^. The enzyme amount catalyzing the decomposition of 1 μmol H_2_O_2_ was used as an activity unit.

Activity of glutathione reductase (EC 1.6.4.2) was determined according to Li [34]. The activity according to the decrease in optical density at 340 nm was monitored (ε = 6.220 × 10^3^ M^−l^ cm^−1^). Activity was expressed in μmol NADPH/(min per mg of protein).

Protein concentration was measured using the modified Bradford method [35].

### 2.12. Statistical Analysis

Statistical analysis was performed using the difference method. This method is used for statistical analysis of the difference between experimental and control replicates cultured under the same conditions, which allows for increasing the significance of differences between the variants and the accuracy of the experiment. For example, when comparing a control (*x*_1_) and an experimental (*x*_2_) sample with *n_i_* repeats, we calculated the difference (*d*) between them by repeats: *n_i_X*_1.1_ − *n_i_X*_2.1_ = *d*_1_; *n_i_X*_1.2_ − *n_i_X*_2.2_ = *d*_2_, etc. Then, we determined the arithmetic mean differences: (*d*_1_ + *d*_2_ + … + *d_ni_*)/*n_i_* = $\bar{d}$.

The deviations *d*–$\bar{d}$ were calculated between each difference and the mean value. These deviations were squared and summed, and their sums $\Sigma {\left(d-\bar{d}\right)}^{2}$ were used to calculate the errors of the differences *Sd* according to the formula:

$$Sd=\sqrt{S{\bar{X}}_{1}{}^{2}+S{\bar{X}}_{2}{}^{2}}.$$

We calculated Student’s actual reliability criterion:

$$t_{1-2}=\frac{\left({\bar{X}}_{2}-{\bar{X}}_{1}\right)}{Sd}.$$

The actual criterion was compared with the theoretical one, which is in the corresponding table by the number of degrees of freedom calculated according to the formula *v* = (*n_i_*1 − 1) + (*n_i_*2 − 1), and conclusions were drawn using the following rule: if the actual Student’s criterion is equal to the theoretical value or greater than it, then the difference between the variants is significant.

## 3. Results

### 3.1. Obtaining Resting G. polyisoprenivorans Cells 135

When the cultivation conditions change, for example, with the depletion of the growth substrate, aging of the culture or the non-optimal ratio of elements such as N and P, the cells from the active growth phase pass into the stationary phase, and then into the phase of death. This process is accompanied by the formation of cyst-like resting cells, the characteristic features of which are the absence of pronounced metabolic activity, thickening of the membranes, rounding of cells, and a decrease in their size. Under laboratory conditions, the formation of resting cells can be induced by simulating the above processes. Previously, various approaches have been used to obtain CLC cells, including (1) short (up to 5 days) incubation of the cultures grown in a rich medium (LB broth) followed by long incubation under statistic conditions; (2) cultivation in a medium with a 5-fold reduced amount of N (to 0.2 g/L) for 1–4 months; (3) imitation of starvation stress: cultivation of cells in LB and transferring them into 0.2 g/L CaCl_2_-supplemented physiological saline (PS, 0.9% NaCl) (pH 7.25) [36]. These conditions work in different ways for the formation of resting cells, depending on the genus of bacteria. In this work, the simplest way to obtain resting cells growing on a rich medium with subsequent long-term storage was used.

#### 3.1.1. Viability of the Vegetative Cells and CLC

The results of CFU counting of a three-day culture of *G. polyisoprenivorans* 135 showed that the number of cells reached 15.9 × 10^11^ mL^−1^. Heating vegetative cells at 40 and 60 °C for 10 min led to an order of magnitude decrease in the number of surviving cells, to 14.5 × 10^10^ and 9.7 × 10^10^ mL^−1^ (*p* ˂ 0.01), respectively. From the data obtained, it can be concluded that a three-day culture grown on LB medium shows a pronounced thermal sensitivity. Although the number of viable cells after heating at 60 °C is quite high, the high thermal stability of *Gordonia* cells is of particular interest. It was shown that mild “heating at 50 °C can achieve 6 log reductions for vegetative cells at low pressures” [37]. As noted by [38], the injury or death of microbial cells occurs under the influence of damaging environmental factors. The death is irreversible. In some cases, cell damage is sublethal in nature, which leads to the restoration of cell growth. It can be assumed that some features of cells of this genus allow them to survive after exposure to sublethal temperature.

After two months of storage, the numerical value of CFU in the resting cell culture changed within the order and was 23.4 × 10^10^ mL^−1^, indicating little cell death during storage. In this respect, the *G. polyisoprenivorans* 135 strain was significantly different from the *Aeromonas hydrophila* strain, in which by the 35th day the CFU/mL number decreased enormously, to 4.5 CFU/mL. At the same time, total and viable cell values did not change significantly, at 4 × 10^8^ and 1.4 × 10^8^ cells/mL after 3 days and 3.0 × 10^8^ and 4.5 × 10^7^ cells/mL after 35 days for total and viable cells, respectively [39].

**Figure 2:**
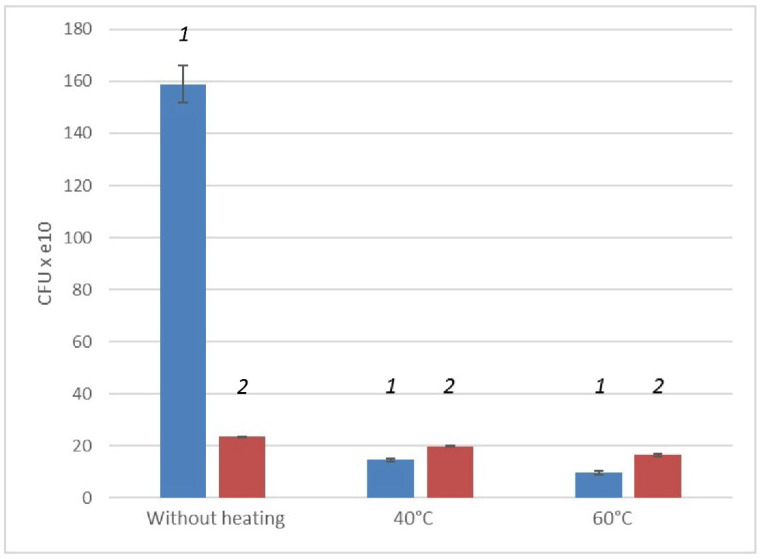
Change in CFU values of a three-day (1) and two-month (2) cultures of *G. polyisoprenivorans* 135 cells under heating.

Heating at 40 °C resulted in a slight decrease in the number of surviving cells to 19.8 × 10^10^ mL^−1^. Heating to 60 °C also led to a decrease in the number of surviving cells; nevertheless, the CFU value was 16.4 × 10^10^ mL^−1^, which was almost two times higher than that for cells treated at this temperature after 3 days of cultivation. Based on the data obtained, it can be concluded that the culture grown in the LB medium shows less pronounced thermal sensitivity after 2 months of storage (Figure 2).

Analyzing the data obtained, it can be concluded that the overall viability of the culture during long-term cultivation, without feeding and reseeding, decreases. However, when cells are stored, their thermal stability increases (Figure 2). This may indicate the appearance of temperature-resistant resting forms in cell cultures during long-term storage.

#### 3.1.2. Morphology and Ultrastructural Organization of *G. polyisoprenivorans* 135 Resting Cells

**Figure 3:**
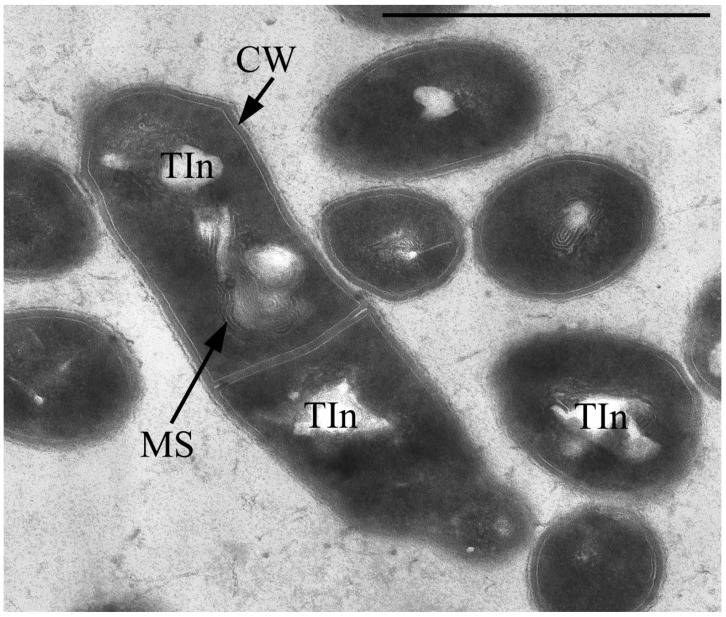
Ultrathin section of *Gordonia polyisoprenivorus* 135 vegetative cells grown in LB. Designation: CW—cell wall; TIn—electron-transparent inclusion; MS—membrane-like structures. Transmission electron microscopy. Bar—1 µm.

The vegetative cells are characterized mainly by rod-shaped cells and can vary in length from 0.8 to 2–3 µm. In addition to the murein layer, the cell envelope has a thin capsular layer. In the central part of the cytoplasm of cells in the region of the nucleoid, large myelin-like (sometimes membrane-like) formations associated with electron-transparent inclusions (which are very similar to the previously described inclusions in the 1CP strain [30]) are often found (Figure 3).

**Figure 4:**
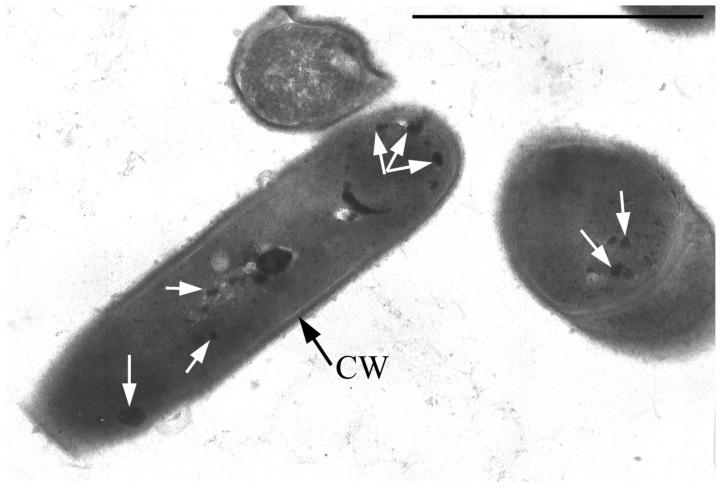
Ultrathin section of *G. polyisoprenivorans* 135 resting cells. Transmission electron microscopy. Designation: CW—cell wall; white arrows indicate electron-dense acidocalcisome-like inclusions.

Single resting cells (RC) of *G. polyisoprenivorans* 135 in the population are represented by short ovoids and rod-shaped cells up to 2 µm in length. RC are characterized by a thicker cell wall compared to vegetative forms, the absence of electron-transparent inclusions in the cytoplasm and a very high content of electron-dense inclusions in the cytoplasm, localized in the periphery, in the central part of the cytoplasm of cells, and on the outer surface of cells (Figure 4). Electron-transparent inclusions of *G. polyisoprenivorans* 135 are similar to inclusions of triacylglycerides of rhodococci. Rhodococci are able to utilize reserved triacylglycerols (TAGs) in the late stationary phase (or in the absence of a carbon source). Apparently, the degradation of reserved TAG is an important process for microbial cells in natural energy-poor environments [40].

**Figure 5:**
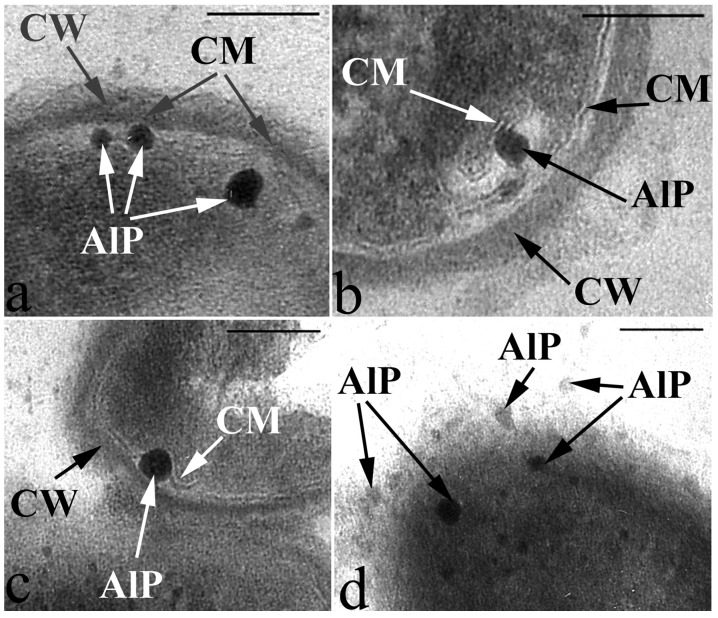
Peculiarities of acidocalcisome-like particles (AlP) localization as they are released from the cell into the environment in resting cells of *G*. *polyisoprenivorans* 135. (**a**) AlP in the cell cytoplasm on the inner surface of the cytoplasmic membrane (CM); (**b**) AlP inside the invagination of the CM on its outer surface; (**c**) AlP in the space between the cell wall (CW) and CM; (**d**) AlP in the extracellular space. Transmission electron microscopy (fragments of ultrathin sections). Designations: CM—cytoplasmic membrane; CW—cell wall; AlP—acidocalcisome-like particules. Bar—100 nm.

The electron-dense inclusions have some similarities with polyphosphate inclusions, but they differ in their dense homogeneous contents and the presence of some limiting membrane (Figure 4 and Figure 5).

**Figure 6:**
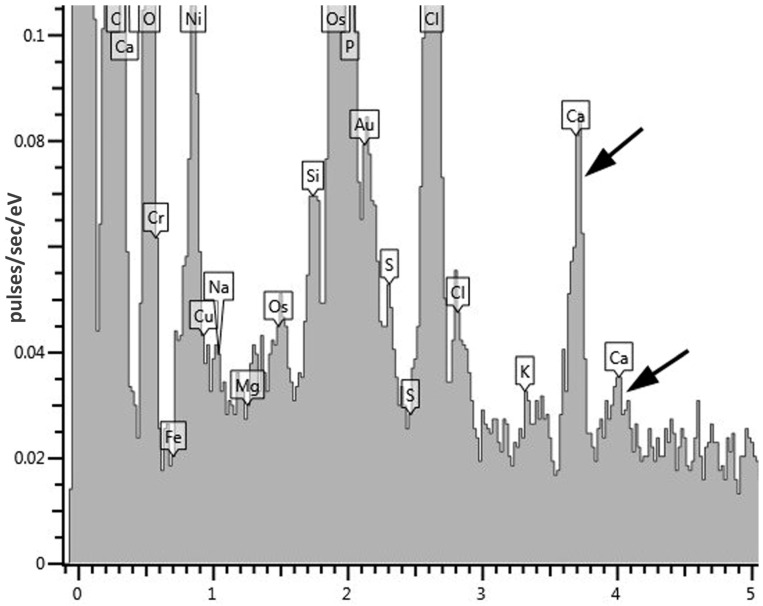
An X-ray spectrum of electron-dense acidocalcisome-like ultrastructures in cyst-like cells of *G. polyisoprenivorans* 135. Arrows indicate calcium peaks.

The study of the composition of these electron-dense inclusions using X-ray microanalysis showed, in addition to the expected peak of phosphorus (P), characteristic of polyphosphate granules, peaks of calcium (Ca) (Figure 6). Based on the morphology and composition, the observed inclusions can be classified as acidocalcisome-like ultrastructures. Acidocalcisomes are inclusions that are quite universal for the living world and are found both in prokaryotes and in a number of eukaryotic organisms.

Acidocalcisomes are specific intracellular electron dense inclusions that are surrounded by a membrane and include polyphosphates (polyP), pyrophosphate (PPi), and various cations, primarily calcium, magnesium and other elements [41].

Interestingly, volutin granules found in the archaea *Methanosarcina acetivorans* and other archaea have the same ultrastructural characteristics and high levels of phosphorus and calcium compounds [42,43,44], and are very similar to the acidocalcisomes of the genera *Agrobacterium* and *Rhodospirillum* bacteria [45,46,47,48].

The main functions of acidocalcisomes are cation and phosphorus storage, which are involved in polyP metabolism, calcium homeostasis and osmoregulation [45].

**Figure 7:**
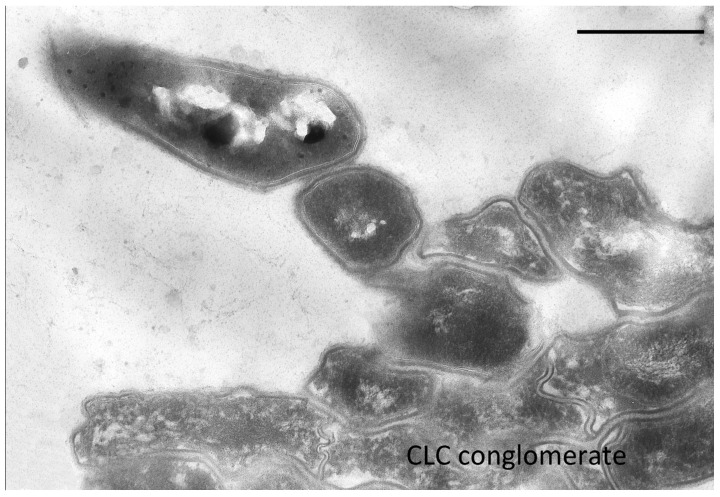
Ultrathin section of CLC conglomerate of *G. polyisoprenivorans* 135 (2 years storage). Bar—1 µm.

In the resting cell population of *G. polyisoprenivorans* 135, multicellular conglomerates are common. Cells in conglomerates differ significantly from single RS. Cell envelopes in conglomerates are much thinner, the cytoplasm is sparse, the ribosomal content is weakly expressed, and inclusions of polyphosphates and acidocalcisome analogs are absent (Figure 7).

#### 3.1.3. Protein Content in Cells and Enzymatic Activity Assay

Analysis of the protein content in cells and enzymatic activity showed that the total amount of protein in cells decreases with storage time. This decrease was most significant during the first 2 weeks of storage. Subsequently, the decline was not so pronounced. In the course of our work, we obtained preliminary data demonstrating that the dynamics of enzymatic activity in resting cells depends on the enzyme function. In actively growing vegetative cells of strain 135, with benzoate as a growth substrate, the activity of catechol 1,2-dioxygenase was 0.850 U/(mg of protein), the activity of catechol 2,3-dioxygenase was absent [49]. It is expected that in cells grown in a rich environment, the activity of benzoate degradation enzymes is very low throughout cell storage, and practically disappears after three months of storage. The detected 1–3 U/(mg of protein) can be considered as the base activity of uninduced cells. In contrast, the activity of catalase, the enzyme responsible for cell protection against oxidative stress increases by the end of the second month of cell storage (Table 1). For the first time, this pattern was observed for cells of *Stenotrophomonas* sp. str. FM3 and *Morganella morganii* subsp. *sibonii* str. FF1 [50]. The transition of cells from vegetative growth to a rest state is not a simple cessation of the metabolic activity of cells. As shown by Abdallah et al. [51], incubation of “marine bacteria *Vibrio parahaemolyticus* and *V. alginolyticus* in seawater for 8 months” resulted in altered biochemical and enzymatic profiles, accompanied by the appearance of minicells and a change in the set of outer membrane proteins in starved cells. *Aeromonas hydrophila* strains “lost adhesive properties and exhibited a different behavior in the expression of *aerA*” after 35 days of storage [39].

Based on the data obtained, it can be concluded that the activity of Cat 1,2-DO and Cat 2,3-DO, as inducible enzymes, when cultivated in a rich medium, is very low and completely disappears after two months of storage. Catalase is one of the main defense enzymes against oxidative stress. Its specific activity in cells passing into a dormant state not only does not decrease, but also increases. Insignificant glutathione reductase activity was found in the cells. Glutathione reductases reduce the disulfide bond of oxidized glutathione to the sulfhydryl form. The concentration of glutathione determines the ability of the cell to inhibit oxidation. In this case, we did not receive confirmation of the role of glutathione reductase in this process in the cells of strain 135.

### 3.2. Peculiarities of Exit from Dormancy of Gordonia polyisoprenivorans 135 Cells

**Figure 8:**
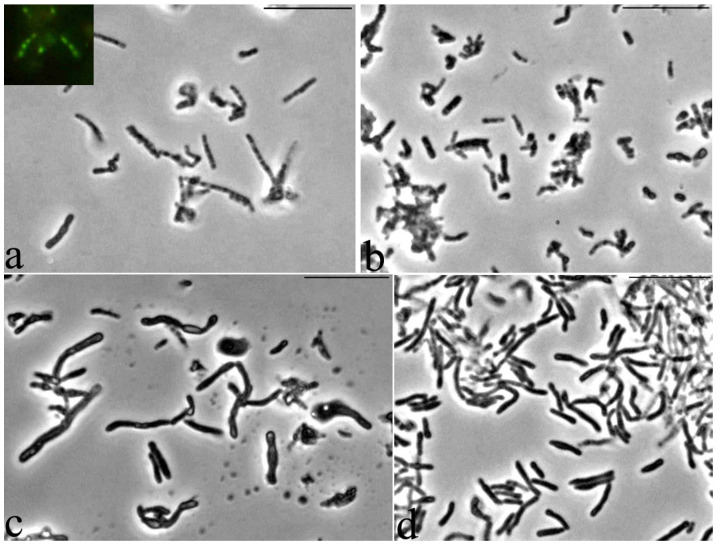
Dynamics of germination of *G. polyisoprenivorans* 135 resting cells. (**a**) 1 h; (**b**) 8–10 h; (**c**) 26 h; (**d**) 48 h. Light microscopy. Phase contrast. Bar—10 µm.

**Figure 9:**
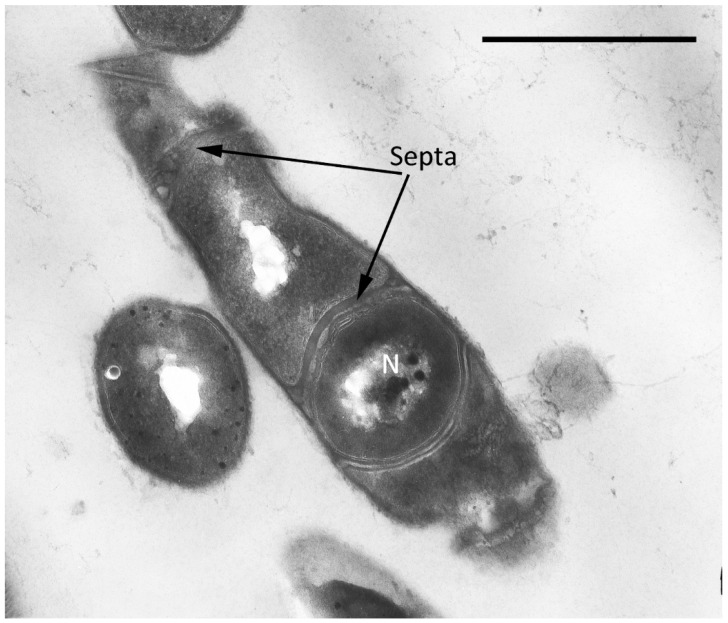
Ultrathin section of CLCs of *G. polyisoprenivorans* 135 with a septum formed inside the cytoplasm (2 years of storage). Designation: N—nucleoid. Bar—1 µm.

It was found that storage for two years of resting cells (RS) of *G. polyisoprenivorans* 135 culture, grown on a medium with a nitrogen limit, led to the formation of large, up to 10 μm, multicellular forms as chains of discrete small cells inside a common shell, having a type of slime layer (Figure 8a). Fluorescence microscopy of this sample using a Live/Dead dye revealed that the chains of small cells in the common shell fluoresced green and were therefore intact and alive (Figure 8a, insertion). According to several authors, actinobacteria slime layers perform such functions as protection against the actions of pH values, temperature, the presence of detergents, biocides, changes in moisture, and ultraviolet exposure that are not optimal for growth, which provides an ecological advantage to the cells and allows them to survive during unfavorable periods of life [52]. However, during long-term (up to two years) storage of the *Gordonia polyisoprenivorans* 135 culture, the transition to the dormant state was apparently accompanied by fragmentation of the mother cell’s cytoplasm into chains of ultra-small spherical forms (Figure 8a). Analysis of ultrathin sections shows the presence of formed intracytoplasmic septa (Figure 9).

An increase in the density of most cells was detected, and small rounded cells appeared in the population 8–10 h after the start of germination, which indicates the beginning of the release of small cells from the general large cell shells of large cell forms (Figure 8b). For *Escherichia coli*, 18 ± 1% of persister cells began to wake within 6 h of cultivation [53]. As the authors noted, the process of renewal of vegetative growth of cells depends on the perception of nutrients, which is based on the work of membrane receptors that transmit a signal to ribosomes through the secondary messenger cAMP. As a result, persistent *E. cells* wake up and use chemotaxis to get nutrients.

The morphology of the cells changed abruptly 26 h after the beginning of germination—there was a significant lengthening and thickening of some of the cells in the population (Figure 8c). After 48 h, almost the entire population was represented by cells of two types: long thick curved rods of uneven thickness and regular rod-shaped cells of medium length (Figure 8d). Apparently, such a morphological division may be the result of phenotypic dissociation in this bacterium, caused by the exit from the dormant state.

In the variant of cell development for 3 days on a rich medium followed by starvation, there was no formation of the slime layer. However, microscopic examination of resting cells showed that in both storage variants, the cells were thin optically dense non-branching rods.

In contrast to the previously studied cultures, *Rhodococcus opacus* 1CP, *Arthrobacter agilis* lush 13, and *Microbacterium foliorum* BN52, the resumption of *G. polyisoprenivorans* 135 cell growth was much slower [25,36,54]. After 24 h of cultivation, no increase in the optical density of the culture in rich medium was observed. After 48 h of cultivation, this indicator reached the values corresponding to 10–14 h after germination of other studied actinobacteria. Nevertheless, microscopic studies of the samples showed that already by 26 h after germination, morphological changes, expressed in significant changes in shape and size, occurred in the cell population during the transition to vegetative growth. The length of some cells reached 10 µm. At the same time, there was no branching of cells typical for rhodococci. Vegetative cultures of *G. polyisoprenivorans* 135 in rich LB medium were used as a control.

**Figure 10:**
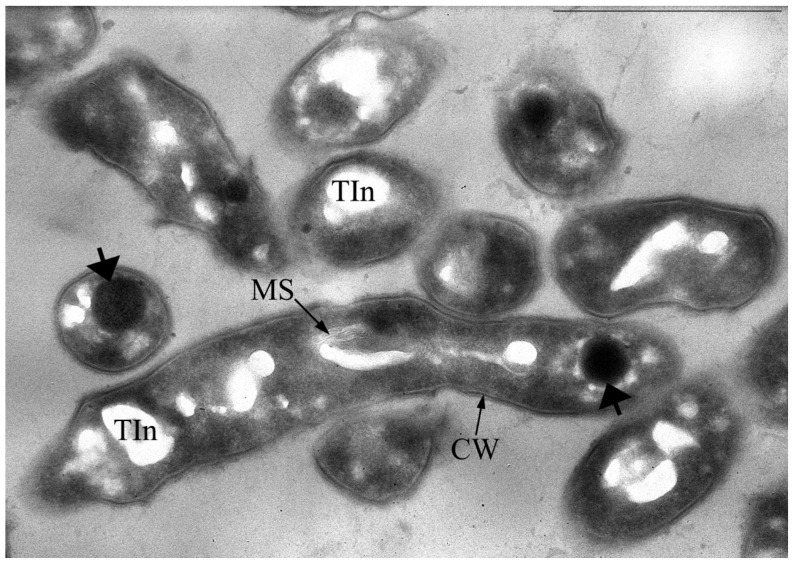
Ultrathin section of *G. polyisoprenivorans* 135 cells 24 h after the start of germination. The appearance of membrane-like structures (MS), reduction in acidocalcisome-like ultrastructures, and the appearance of large polar electron-dense spherical inclusions (indicated by thick arrows) are detected. Transmission electron microscopy. Ultrathin section. Designations: CW—cell wall; Tin—electron-transparent inclusion; MS—membrane-like structures. Bar—1 µm.

Electron microscopic analysis of ultrathin sections of cells germinating after dormancy showed that 24 h after the start of germination, the cells practically lost their acidocalcisome-like ultrastructures (Figure 10). This indicates their active role in the processes of metabolic and ultrastructural rearrangements during cell exit from the resting stage.

### 3.3. Activation of Benzoate Utilization Enzymes in G. polyisoprenivorans 135 Cells

The resumption of the active growth of cells occurred not only when they were transferred to a fresh rich medium. The addition of a mineral medium with sodium benzoate as the only growth substrate also led to the resumption of active cell growth. This growth was accompanied by the induction of benzoate degradation enzymes. As noted earlier (Table 1), in cells grown in a rich medium, the activity of the key benzoate degradation enzymes, Cat 1,2-DO and Cat 2,3-DO, does not exceed a few U/(mg of protein). The addition of benzoate to cells stored for 3 days in rich media resulted in a rapid increase in Cat 1,2-DO activity. After 5 h of induction, the specific activity increased to twice that of non-induced cells. Cells after 2 months of resting required more time to reach such activity indices (Table 2). By the fifth day, the enzyme activity was approximately the same in cells with different storage periods. Previously, it was shown that the benzoate degradation by *G. polyisoprenivorans* 135 cells proceeds with the formation of catechol and its cleavage in the *ortho*-position [28]. The specific activity of catechol 1,2-dioxygenase in induced cells was approximately four times lower than in cells of the same strain adapted to benzoate [49]. However, this difference is not surprising, since several cell passages are necessary to obtain maximum enzyme activity. No activity of enzymes for ring cleavage at position 2,3-(*meta* cleavage) was observed. In cells after resting, the activity of Cat 1,2-DO was detected and no activity of Cat 2,3-DO was detected (Table 2). Thus, resting did not affect the pattern of enzyme induction.

### 3.4. Dynamics of BDO Activity in G. polyisoprenivorans 135 Cells during Vegetative Growth-Rest-Vegetative Growth Transition

**Figure 11:**
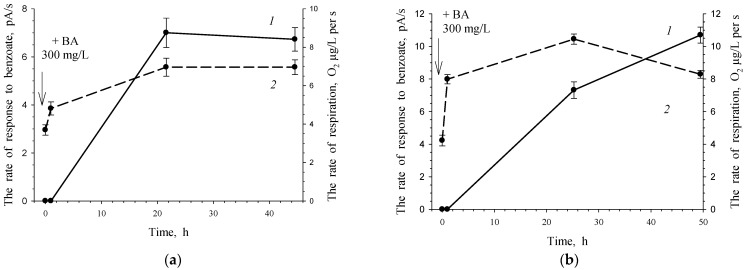
Response of *G. polyisoprenivorance* 135 cells toward benzoate under non-growth conditions. (**a**) *1*—Activity of BDO toward 3.47 mM benzoate; *2*—consumption of O_2_ without a substrate. (**b**) Response of the same cells to a second portion of benzoate 7 days after the first one. *1*—Activity of BDO toward 3.47 mM benzoate, *2*—consumption of O_2_ without a substrate.

Under non-growth conditions, both an increase in cell respiration and an increase in BDO activity occurred in response to benzoate’s addition to the cell suspension (Figure 11a). Repeated addition of the substrate after a week of cell suspension storage resulted in a higher cell response for both indicators. To activate resting cells, the substrate, benzoate, was used at approximately the same concentrations as in the experiments for cell activation under non-growing conditions (250–300 mg/L).

In the case of growth conditions after 5 days of cultivation, Cat 1,2-DO activity remained at a high level, 0.156 U/(mg of protein). It can be assumed that under non-growth conditions, this enzyme is retained in cells for rather a long time. The cell’s response to the reintroduction of the substrate after another week is more intense due to the presence of catabolic enzymes, which determines a shorter lag phase and higher enzymatic activity (Figure 11b).

*G. polyisoprenivorans* 135 cells grown in LB medium and immobilized on a carrier retained metabolic activity when stored for a year at 4 °C (data not shown). The response of immobilized cells to the substrate in this case was due to the processes of substrate transport into actinobacterial cells. In this case, the microbial cells of the receptor element during the measurements were “fed” with benzoate during the reaction. Noteworthy is the fact that the storage of immobilized cells between measurements during the first 4 months (between 30 and 150 days) led to a slight decrease in cell activity in relation to benzoate. One day later (after checking the reaction, i.e., after the benzoate entered the cells), the activity of the cell reaction reached almost the same level.

Subsequent storage for 4 months (151–280 days) resulted in the loss of half of the cell activity; however, the level of response to benzoate remained at a high level. The last two points of the experiment reflect the dynamics of the behavior of immobilized cells when they are stored on an electrode in distilled water. The pool of endogenous substrates that provide the cell’s energy consumption probably decreased, which was accompanied by a decrease in the cell response from 138.83 pA/s (279 days) to 25.79 pA/s (the last point—286 days).

**Figure 12:**
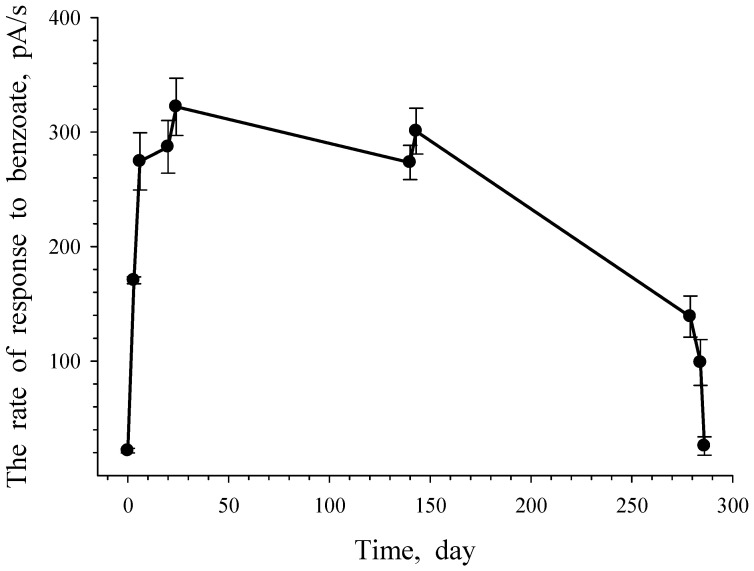
Response of *G. polyisoprenivorance* 135 immobilized cells toward benzoate. Each dot reflects the rate of oxygen consumption with the injection of benzoate.

The results of this experiment show that immobilized cells under periodic supply of the substrate retain their metabolic activity for a long time and are capable of a quick response when a new portion of the substrate is supplied to them (Figure 12). The revealed features of this non-spore-forming culture indicate a high adaptability of cells to survive unfavorable environmental conditions and a fast restructuring of metabolism when a growth substrate appears.

**Figure 13:**
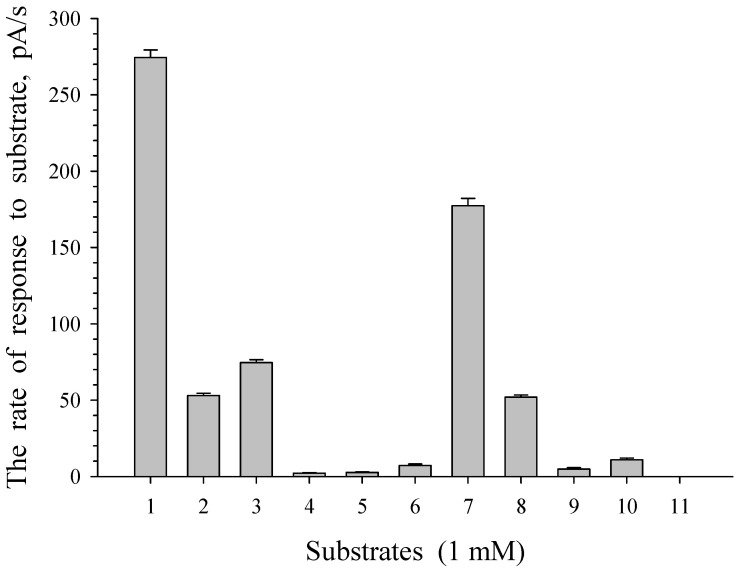
Substrate specificity of immobilized cells of *G. polyisoprenivorance* 135. 1—Benzoate, 2—sucrose, 3—glucose, 4—arabinose, 5—xylose, 6—maltose, 7—acetate, 8—pyruvate, 9—citrate, 10—succinate, and 11—salicylate.

The substrate specificity of *G. polyisoprenivorans* 135 cells grown on LB medium and immobilized on a carrier was studied. Under these conditions, the appearance of the response of cells to the substrate was associated primarily with the activation of substrate transport processes into the immobilized cells. The study showed that the highest cell response was obtained to add benzoate as a growth substrate. The culture response to acetate, glucose, sucrose, and pyruvate was about half that to benzoate (Figure 13).

This corresponds to the behavior of actinobacterial cells as a representative of dispersal microflora, or oligotrophic microflora, which is characterized by a low requirement for nutrients and the use of monomers formed by other microorganisms. It is known that actinobacteria can use very low substrate concentrations in nature [55].

Earlier studies have shown that oligotrophic growth kinetics is characteristic of rhodococci utilizing oil hydrocarbons [56,57,58]. It manifests itself in low growth rates, low energy consumption for maintenance needs, and high affinity for the substrate, which helps to increase the consumption of nutrients without changing the cell surface area, the ability to increase the surface area to volume ratio due to cell fragmentation and the presence of cell branching assimilating the hydrocarbon substrate. The ability of bacteria to undergo oligotrophy is explained by the structure of their membranes, which contain many membrane-associated proteins that determine the diversity of transport systems in these bacteria [59]. These bacteria can switch from feeding on oil hydrocarbons to carbohydrate metabolism. At the same time, rhodococci are not capable of growth at high concentrations of hydrocarbons.

Resting cells are effectively used to carry out catalytic reactions to obtain target products. As noted by de Carvalho [60], resting cells are grown to a certain optical density, washed from the rest of the medium by the water or buffered solution, and resuspended in the desired buffer for biocatalysis. As a result of such processing, unused growth substrate and nutrients, as well as unwanted growth metabolites, are removed from the cell suspension. This promotes higher formation of the target product and facilitates the recovery of the product. Resting cells of *Halomonas elongata* DSM 2581^T^ were used to develop a technology and significantly increased the yield of vanillic acid from ferulic acid [61]. However, in this case, cells were used that were not subjected to long-term storage. The present study shows the use of dormant cells for the decomposition of aromatic compounds, for example, benzoate, which is provided by the induction of degradation enzymes and is based on the ability of cells to quickly pass from a dormant state to active growth.

## 4. Conclusions

Thus, the data obtained in the present work show that *G. polyisoprenivorans* 135, which is a destructor of several toxic compounds, can survive under unfavorable conditions due to the formation of specific dormant cell forms (cyst-like cells) and cell conglomerates. This ability reflects the general tendency of non-spore-forming bacteria to remain viable under stress conditions. A characteristic feature of *G. polyisoprenivorans* 135 is its long-term preservation of metabolic activity in an immobilized state, a quick response to the introduction of a growth substrate against the background of a relatively long period of dormancy in comparison to other representatives of actinobacteria. Cell germination is accompanied by the formation of two populations of the cells, differing in size. This probably allows the cells to be introduced into different habitats, and the oligotrophy inherent to bacteria of this group gives them a competitive advantage over bacteria of other classes.

## Figures and Tables

**Table 1:** Enzymatic activity of vegetal and resting cells of bacteria *G. polyisoprenivorans* (sample, the cells were cultured with stirring for three days, and then stored under stationary conditions).

Cultivation Period	V, mL	Protein Content in Cell-Free Extracts, mg/mL	Cat 1,2-DO Activity, U/mg of Protein	Cat 2,3-DO Activity, U/mg of Protein	Catalase Activity, U/mg of Protein	Glutathione Reductase Activity, U/mg of Protein
3 days	4.1	5.0 ± 0.2	0.0023 ± 0.0006	0.0008 ± 0.0004	0.0043 ± 0.0006	0.0038 ± 0.0008
2 weeks	8.8	1.6 ± 0.1	0.0031 ± 0.0008	0.0006 ± 0.0002	0.0564 ± 0.0012	0.0044 ± 0.0012
1 month	7.1	1.6 ± 0.2	0.0034 ± 0.0007	0.00008 ± 0.00004	0.1445 ± 0.0007	0.0131 ± 0.0039
2 months	5.1	2.2 ± 0.1	0.0022 ± 0.0004	0.00011 ± 0.00003	0.1636 ± 0.0012	0.0029 ± 0.0004
3 months	4.4	2.5 ± 0.1	0	0	0.1435 ± 0.0009	0.0021 ± 0.0008
6 months	3.6	3.1 ± 0.2	0	0	0.0038 ± 0.0004	0.0024 ± 0.0002

**Table 2:** Ability of *G*. *polyisoprenivorans* 135 culture to induce biodegradation enzymes after resting.

Cell Storage Time in LB Medium	Cultivation Time in Medium with Benzoate	Protein Content in Cell-Free Extracts, mg/mL	Cat 1,2-DO Activity, U/mg of Protein	Cat 2,3-DO Activity, U/mg of Protein
3 days	5 h	0.51 ± 0.03	0.0360 ± 0.008	0.0018 ± 0.0004
2 months		0.48 ± 0.02	0.0022 ± 0.0005	0.0010 ± 0.0003
3 days	1 day	1.24 ± 0.06	0.178 ± 0.006	0.0023 ± 0.0007
2 months		0.82 ± 0.01	0.098 ± 0.003	0.0021 ± 0.0002
3 days	5 days	2.17 ± 0.01	0.155 ± 0.002	0.0340 ± 0.0105
2 months		1.64 ± 0.05	0.147 ± 0.005	0.0280 ± 0.009

## Data Availability

The data presented in this study are available on request from the corresponding author.
